# Decisions Parents Make When Faced With Potentially Life-Limiting Fetal Diagnoses and the Importance of Perinatal Palliative Care

**DOI:** 10.3389/fped.2020.574556

**Published:** 2020-10-22

**Authors:** Krishelle L. Marc-Aurele

**Affiliations:** Division of Neonatology, Department of Pediatrics, University of California, San Diego, San Diego, CA, United States

**Keywords:** perinatal palliative care (PPC), decision-making, pregnancy, life-limiting fetal diagnoses, decisions

## Abstract

When parents face a potentially life-limiting fetal diagnosis in pregnancy, they then have a series of decisions to make. These include confirmatory testing, termination, and additional choices if they choose to continue the pregnancy. A perinatal palliative team provides a safe, compassionate, and caring space for parents to process their emotions and discuss their values. In a shared decision-making model, the team explores how a family's faith, experiences, values, and perspectives shape the goals for care. For some families, terminating a pregnancy for any reason conflicts with their faith or values and pursuing life prolonging treatments in order to give their baby the best chances for survival is the most important. For others, having a postnatal confirmatory diagnosis of a life limiting or serious medical condition gives them the assurance they need to allow their child a natural death. Others want care to be comfort-focused in order to maximize the time they have to be together as a family. Through this journey, a perinatal palliative team can provide the support and encouragement for families to express their goals and wishes, as well as find meaning and hope.

## Introduction

During pregnancy, ultrasounds and blood test results in the first two trimesters provide information about the fetus' health. When these results suggest a potentially life-limiting fetal diagnosis, parents are faced with a series of decisions. The first choices involve whether to obtain more diagnostic information via invasive or non-invasive tests during the pregnancy. Invasive testing includes chorionic villus sampling or amniocentesis in order to check for genetic mutations or aneuploidy. Parents need to weigh the risks of invasive testing against their desire for more information and greater certainty to help them with time-sensitive decisions. Although rates of pregnancy loss have been difficult to estimate, a recent meta-analysis suggests no significant risk of miscarriage with chorionic villus sampling and a miscarriage risk of 1:300 with amniocentesis (1). There are other risks associated with invasive testing, however, including bleeding, Rh sensitization, rupture of membranes, and infection (2). The recommended time frame to perform these invasive tests, between 10 and 20 weeks' gestation, is exactly when concerns about the fetus are raised and therefore a relatively narrow window of time in which to make decisions about testing (3). Diagnostic results may take up to 1–2 weeks to return. Although prenatal genetic testing has been expanded to include exome sequencing, it unfortunately cannot identify all possible abnormalities. Despite these limitations, the results may determine whether some families continue the pregnancy.

As an alternative, parents may first choose to decline invasive testing and opt for non-invasive testing which includes cell-free DNA screening and additional imaging. Cell-free DNA screening has a high sensitivity and specificity for Trisomy 21 and Trisomy 18, but lower performance for Trisomy 13, other chromosomal anomalies, and microdeletions (4). If non-invasive cell-free DNA screening is suggestive of a genetic abnormality, invasive testing is still needed to confirm the diagnosis before termination. Additional imaging includes fetal echocardiogram, three-dimensional fetal ultrasound, or fetal magnetic resonance imaging. For advice and opinion on the suspected diagnosis and outcomes, parents may also want to consult with pediatric subspecialists. What adds to the complexity of the situation is that maternal fetal medicine specialists have two patients: the mother and the fetus. Their duty is to separate information about the risks and benefits of various choices for both patients, recognizing that decisions made will have linked outcomes (5). As such, the maternal fetal medicine specialist will also review for the mother potential pregnancy complications associated with fetal anomalies: preeclampsia, preterm labor, polyhydramnios, and mirror syndrome (6–10).

After gathering as much data as possible, parents will consider whether or not to terminate the pregnancy. This decision often must be made within days or a couple of weeks after learning about a fetal abnormality. While many technologies offer to improve diagnostic certainty or prognostication, parents need to know about limitations in predicting survival and outcome variability. Each test, imaging modality, and consultation is an option for parents to pursue or decline. For some families, terminating a pregnancy is not consistent with their faith or values so undergoing invasive or non-invasive testing to inform a decision to terminate does not make sense. It is possible that a life-limiting fetal diagnosis may not be discovered until after termination is no longer an option, but families may still want information to prepare themselves for possible outcomes. Other families may feel the degree of uncertainty associated with some information acquired during pregnancy is not worth the additional stress. And some families want more time to process the news before obtaining more data.

## Perinatal Palliative Consultation

“Perinatal palliative care refers to a coordinated care strategy that comprises options for obstetric and newborn care that include a focus on maximizing quality of life and comfort for newborns with a variety of conditions considered to be life-limiting in early infancy. With a dual focus on ameliorating suffering and honoring patient values, perinatal palliative care can be provided concurrently with life-prolonging treatment” (11). There is a misconception that palliative care applies only to cases where the goals are to allow a natural death. In fact, perinatal palliative teams advocate primarily for respect of parental wishes, supporting a spectrum of goals from comfort-focused to life-prolonging care. A perinatal palliative team is typically multidisciplinary including a physician, nurse, social worker, and spiritual counselor (12). Some palliative teams are imbedded within fetal diagnostic centers, enabling simultaneous palliative consultation (13). For centers without integrated palliative teams, however, there are barriers to early referral and palliative consultation might not occur for weeks (14). Because decisions to terminate often need to occur relatively quickly, families who chose termination are likely to miss the opportunity for perinatal palliative consultation. In practice, consultation with a perinatal palliative team most frequently occurs after parents have declined termination with plans instead to continue the pregnancy (15). No matter which decision parents make (i.e., to terminate or continue the pregnancy), however, all parents experience losses, find themselves planning for a future they did not hope for or expect, and can benefit from the additional support for decision-making that perinatal palliative teams can provide (16).

Providing support to parents as they make decisions is central to the perinatal palliative consultation. Amidst the variable and sometimes overwhelming emotions that parents can experience, the perinatal palliative team allows parents to explore their goals in order to promote shared decision-making with regard to obstetric management as well as postnatal care for the baby. Mixed, changing, and sometimes conflicting feelings of shock, concern, disbelief, denial, anger, love, shame, hope, and guilt are normal for parents to experience when hearing serious news about their baby (17). The perinatal palliative team fosters a safe, compassionate, and supportive environment for parents to process their emotions and discuss their values (18). Without the ability to address their feelings or spiritual distress, parents may not be ready to explore their hopes for their baby's care, let alone make changes to the plan of care. For example, a spiritual counselor on the perinatal palliative team is especially important if a parent is worried about the “right” thing to do with respect to her faith. Addressing the meaning of the situation in the context of her faith is necessary before approaching decision-making about treatment options for the baby. By validating and reflecting what parents express and in turn building trust, the perinatal palliative team can support a parent's voice in decision-making and facilitate communication between parents and other medical teams (19).

## Birth Plans

One way in which the perinatal palliative team supports a parent's voice and decisions is to collaborate on a birth plan. A birth plan is similar to an advanced directive for the pregnancy, delivery, and neonatal care. By sharing their birth plan with the medical team, families create additional opportunities for informed and shared decision-making. A birth plan documents parental hopes, wishes, and goals of care, serving as a communication tool for the entire medical team. Personalized birth plans vary in content but generally address the following components: pain control for the mother, preference for fetal monitoring, mode of delivery, who to be present in the delivery room, resuscitation measures desired at birth, medical management for the baby, as well as wishes for memory making, and ceremonies (20). Table 1 presents suggested topics for a birth plan. Writing a birth plan allows parents to identify and articulate goals ahead of time before a potentially overwhelming emotional moment. Parents have reported that creating and using a birth plan gave them a sense of control, was therapeutic, and helped them feel prepared (21).

**Table 1:** Suggested topics for a birth plan.

Plan of care for mother
Maternal information including medical record number and date of birth
Preference for fetal monitoring and mode of delivery
Requested persons to be present in the delivery room
Music choices for labor and delivery
Requests for any limitations in medical personnel
Pain control preferences
Request for information regarding maintenance and suppression of lactation
Plan of care for baby
Planned name
Designated person to cut the umbilical cord
Resuscitation measures desired at birth
Goals for medical management for the baby
Code status
Anticipated location for care
Expectations for respiratory and nutrition support
Desired diagnostic testing, imaging, consultations
Symptom management
Any limitations for routine newborn care (i.e. screening and prophylaxis)
Selected persons to be present with the baby
Memory making including caregiving activities, photography, and creation of mementos
Desired spiritual care or ceremonies
End-of-Life Plan
Plans for home hospice care
Organ donation
Autopsy
Preference regarding use of a cooling bassinet
Final Arrangements
Requests for bereavement counseling resources

The most challenging sections of the birth plan to complete usually address fetal monitoring, mode of delivery, and goals of care for baby's management. The 2019 ACOG Committee Opinion on Perinatal Palliative Care states that, “Decisions regarding the appropriateness of intrapartum fetal monitoring in cases like this should be individualized” (11). Some families are willing to accept the possibility of their baby's death during unmonitored labor in order to avoid the operative risks of cesarean section. Other families would rather have fetal monitoring during labor and if necessary, cesarean delivery for fetal distress, to increase the chances for their baby to be born alive. For example, some may want the opportunity for the entire family to meet the baby or to perform a ceremony at birth. For some families, having a postnatal confirmatory diagnosis of a life-limiting or serious medical condition gives them the assurance they need to allow their child to have a natural death. And other families may want to pursue life-prolonging treatments in order to give their baby the best chances for survival.

The uncertainty of whether the baby will survive the pregnancy and birth can make planning for the neonatal period challenging. Some families are ready to consider in detail the choices for baby's care after birth while other families want to defer those decisions until after the baby is born. The perinatal palliative team can help to explain how care for the newborn can be adapted to align with the goals of care. For example, families may want a time-limited trial of respiratory and nutrition support in order to see if the baby can breathe or eat on her or his own. If the baby is unable to breathe independently or feed by mouth safely, then families will need to consider whether providing long-term ventilation or artificial nutrition aligns with their goals for care. As it was during pregnancy, each test, imaging modality, and consultation for the baby is an option for parents to pursue or decline.

The birth plan will vary based on a family's faith, values, and experiences. For example, some families will want specific spiritual care or ceremonies after birth to honor their faith. But there are also some universal themes (22). Janvier et al. found that among families with children who lived with Trisomy 13 or 18, there were “common hopes to bring their child home, give their child a good life, and be together/a family” (23). Parents often have an intrinsic need to feel that they have “done the best that they could” for their child and want to avoid having regrets in the future (24). Maintaining hope is an important source of strength for parents when facing difficult situations (25). A parent's hope can change with time and new information, from hoping for a normal pregnancy, to hoping the baby will be healthier than predicted, to hoping that the baby will be alive at birth to meet the family. During goals of care planning, the perinatal palliative team can encourage and help families to maintain hope as circumstances change. Nearing the day of delivery, parents may worry about future regrets in choosing comfort-focused care, and want time-limited trials of therapy until the baby's diagnosis is confirmed. It is important to validate that concerns about regret can inform the goals of care and to acknowledge that allowing time to pass might reduce parental decision regret (26). There is also the possibility that at birth, the baby's presentation may not match the prenatal diagnosis thereby suggesting a different prognosis. Health care providers need to be flexible and remember that the birth plan serves as a guide for both mother and baby care providers, but it can be modified at any point before and after delivery. Ultimately, what is most important is that parents are supported, ideally by perinatal palliative teams alongside other medical teams, to discuss and express the rationale behind their decisions.

## Memory Making

Whether the goals of care are focused on life-prolongation or comfort, most families whose baby may have a short life want to plan for memory making. The perinatal palliative team offers expertise and experience to support parents in the many ways they want to make memories. Memory making includes “any intervention or experience that encourages contact or interaction between the parents and newborn and any intervention that results in the creation or collection of mementos” (27). Mementos include molds of the baby's hands and feet, foot and handprints, blankets, hats, and clothing that the baby touched, as well as crib cards, hospital bands, and other personal items that were associated with the baby in the hospital. Now I Lay Me Down to Sleep is a non-profit international organization which offers free professional retouched photography to families who have a baby with a life-limiting condition (28). Families may want to take their own photographs or hire a photographer instead. Although parents may not want to see photos of their child right away, the portrait sessions give them the opportunity to have documentation of their child's life available for when they are ready. After the death of an infant, interviewed families emphasize the importance of having as many parenting experiences as possible to reflect upon in bereavement (29). Opportunities for caregiving can include bathing, dressing, and holding their baby as well as talking, singing, and playing music with their baby (30). Families recall that they needed encouragement and guidance to overcome their hesitation in order to spend time with their newborn (31). Some families introduce their baby to siblings and relatives to officially welcome him or her into the family. Making memories can not only validate the importance of the newborn's life and death but also create a sense of identity for individuals as parents, siblings, and grandparents.

## Comfort Focused-Care

If families choose comfort-focused care for their baby, there may be an opportunity to continue the caregiving at home with hospice support. A hospice physician and team provide expertise in end-of-life care at home and ongoing bereavement counseling for the immediate family. A designated pediatrician is customarily not needed unless the goals of care change to include life-prolonging interventions. If the baby is unlikely to survive the trip home, some parents may want to room-in with the baby in the hospital while some families may not have the financial or logistical capacity to room-in. The perinatal palliative team can provide inpatient support to the primary neonatal team who will attend to the baby's pain and symptom management as well as support the family through the dying process. Many parents have little to no experience with death and will want to know what their baby will look like and feel in the last hours of living (32). Generally, babies in the dying process will become less active, sleeping more as time goes on. Any ability to swallow saliva will become impaired. With less intake, decreased urine and stool output is expected. Cool hands and feet along with mottling of the skin is normal. Less commonly, newborns may need treatment for seizures in the dying process. Eventually, when the baby is no longer conscious, periods of apnea, or Cheyne-Stokes pattern respirations will occur. Ordinary techniques to soothe babies, such as holding, rocking, and swaddling, are usually successful to minimize discomfort. Occasionally, morphine or lorazepam may be needed for symptom management of pain or agitation, respectively. Normalizing the signs of dying for families may reduce their anxiety and distress when the time comes. It is not possible to predict when death will occur, but it is possible to prepare families for the unpredictability of death. Using words such as “weeks not months,” “days not weeks,” or “hours not days,” can be helpful (33).

If medically possible, families can choose organ or tissue donation to create a hopeful and positive legacy. Organ procurement teams have specialized training and are the preferred communicators to maintain ethical and clinical standards while initiating organ donation discussions. Referrals to organ procurement organizations should be made in a timely manner when death is anticipated and may occur before birth (34). In addition, some families may agree to a full or limited autopsy to inform subsequent pregnancies or to improve medical knowledge for future families. Others may decline autopsy because they do not need more information, or their faith and values are not congruent with allowing an autopsy. Families may choose final arrangements based on family or faith traditions as well as their budget. Generally, cremation is less expensive than burial but the cost can vary by mortuary and location (35). Some parents will want to designate a relative or friend to do research and get quotes on available services and some families will prefer to do this work on their own.

After a baby dies, some families will want extended time with the baby and may want to use a cooling bassinet to decrease the rate that the body deteriorates (36). Others will say goodbye a few hours after the baby dies. There are physical and emotional aspects of lactation that need to be addressed. Some mothers will want to suppress breast milk production but need education on how to avoid painful engorgement and mastitis. Others will want to continue expressing and donate breast milk as a way to help other families and find meaning in the context of their loss and grief (37). It is important to recommend grief resources and counseling for families whether they go home with hospice or stay in the hospital setting. Some families may prefer to visit online bereavement resources first and others will want to start mental health therapy immediately (38, 39). The perinatal palliative team continues to support families as they make choices to honor their baby that may include a funeral, celebration of life, and donations to charities or research. Additionally, the team often maintains contact with families in bereavement. It is important to share with families that there are no right or wrong ways to grieve or feel when a baby dies.

## Conclusion

Through the journey with a potentially life-limiting fetal diagnosis in pregnancy, a perinatal palliative team can provide the encouragement families need to make decisions based on their faith, values, and experiences. The team has the expertise to create a safe, compassionate, and caring environment for parents to process a number of intense emotions and discuss their values. Although parents must confront uncertainty and complex decision-making for both the mother and baby, perinatal palliative teams support parents to express their goals as well as find meaning and hope.

## Author Contributions

KM-A agrees to be accountable for and conceived, drafted, revised, and approved all aspects of the work.

## Conflict of Interest

The author declares that the research was conducted in the absence of any commercial or financial relationships that could be construed as a potential conflict of interest.
